# The prognostic value of pretreatment inflammatory and nutrition-related indices in patients with cardia cancer

**DOI:** 10.3389/fnut.2025.1559892

**Published:** 2025-11-19

**Authors:** Wenjuan Guo, Jinwei Zhao, Ning Li, Jing Wang

**Affiliations:** 1 Department of Radiation Oncology, Shanxi Province Cancer Hospital/Shanxi Hospital Affiliated to Cancer Hospital, Chinese Academy of Medical Sciences/Cancer Hospital Affiliated to Shanxi Medical University, Taiyuan, Shanxi, China; 2 Department of Pharmacy, Shanxi Province Cancer Hospital/Shanxi Hospital Affiliated to Cancer Hospital, Chinese Academy of Medical Sciences/Cancer Hospital Affiliated to Shanxi Medical University, Taiyuan, Shanxi, China

**Keywords:** pre-treatment, inflammatory index, nutrition index, cardia cancer, overall survival

## Abstract

**Objective:**

To evaluate the prognostic significance of inflammatory and nutrition-related indexes in patients with cardia cancer prior to treatment.

**Methods:**

A total of 229 patients with cardia cancer, diagnosed pathologically and admitted to Shanxi Cancer Hospital between January 2017 and December 2018, were included. Statistical analysis was conducted using SPSS 26.0, and the optimal cut-off values for body mass index (BMI), peripheral blood platelet-to-lymphocyte ratio (PLR), hemoglobin-to-erythrocyte distribution width ratio (HRR), prognostic nutritional index (PNI), and integrating hemoglobin, albumin, lymphocyte, and platelets (HALP) score were determined with X-tile 3.6.1 software. Survival analysis was performed using the Kaplan–Meier method, with variability assessed by the Log-rank test. Univariate and multivariate Cox proportional hazards regression analyses were used to evaluate the prognostic value of the variables. A prognostic risk stratification model was developed, incorporating age, tumor node metastasis classification (TNM), treatment options, HRR, and PNI, to classify patients into low-risk, intermediate-risk, high-risk, and very high-risk groups. The predictive value of the model was assessed using receiver operating characteristic (ROC) curves.

**Results:**

The optimal critical values of BMI, PLR, HRR, PNI, and HALP score were 20.43 kg/m^2^, 140.59, 2.85, 49.98, and 28.23, respectively. Cox univariate analysis showed that age, TNM stage, treatment regimen, BMI, PLR, HRR, PNI, and HALP score were correlated with the prognosis of patients with cardia cancer (*p* < 0.05). The results of Cox multifactorial analysis showed that age, TNM stage, treatment regimen, HRR, and PNI were independent factors affecting the prognosis of patients with cardia cancer (*p* < 0.05). Age ≥ 60 years, TNM stage III/IV, treatment regimen without surgical involvement, HRR < 2.85, and PNI < 49.98 were considered as risk factors. These five variables were assigned points based on their HRs. The patients were categorized as low-risk (0–6 points), intermediate-risk (7–8 points), high-risk (9 points), and very-high-risk (10–11 points) groups. The median survival times were undefined, 18.05 months, 15.63 months, and 9.10 months, respectively, with statistically significant differences among the four groups (*p* < 0.05). ROC curve analysis, the area under the curve (AUC) of the prognostic risk stratification model was 0.80, which was higher than those for age (0.56), TNM stage (0.63), treatment regimen (0.73), HRR (0.57), and PNI (0.63).

**Conclusion:**

Pre-treatment inflammatory and nutrition-related indexes, HRR and PNI, were closely associated with the prognosis of patients with cardia cancer. Combining age, TNM stage, treatment regimen, HRR, and PNI for prognostic risk stratification could significantly enhance the accuracy of prognostic predictions.

## 1 Introduction

Cardia cancer is a prevalent digestive tract tumor with a rising incidence over recent years (1). Despite the use of radical surgery for early-stage cardia cancer, some patients exhibit poor therapeutic outcomes and limited long-term survival due to rapid tumor metastases (2). Therefore, identifying more effective predictive biomarkers beyond the current tumor node metastasis (TNM) staging system is crucial for improving clinical outcomes. Studies have demonstrated that systemic inflammatory response and nutritional status indicators play a significant role in tumor progression and prognosis, influencing the outcomes of various malignant tumors (3–5). To examine the impact of inflammation and nutrition-related indexes on the prognosis of patients with cardia cancer, we conducted a retrospective analysis of the association between pretreatment body mass index (BMI), peripheral blood platelet-to-lymphocyte ratio (PLR), hemoglobin-to-erythrocyte distribution width ratio (HRR), prognostic nutritional index (PNI), and HALP score (consisting of hemoglobin, albumin, lymphocyte, and platelet count) with patient outcomes.

## 2 Patients and methods

### 2.1 Patients

**Figure 1:**
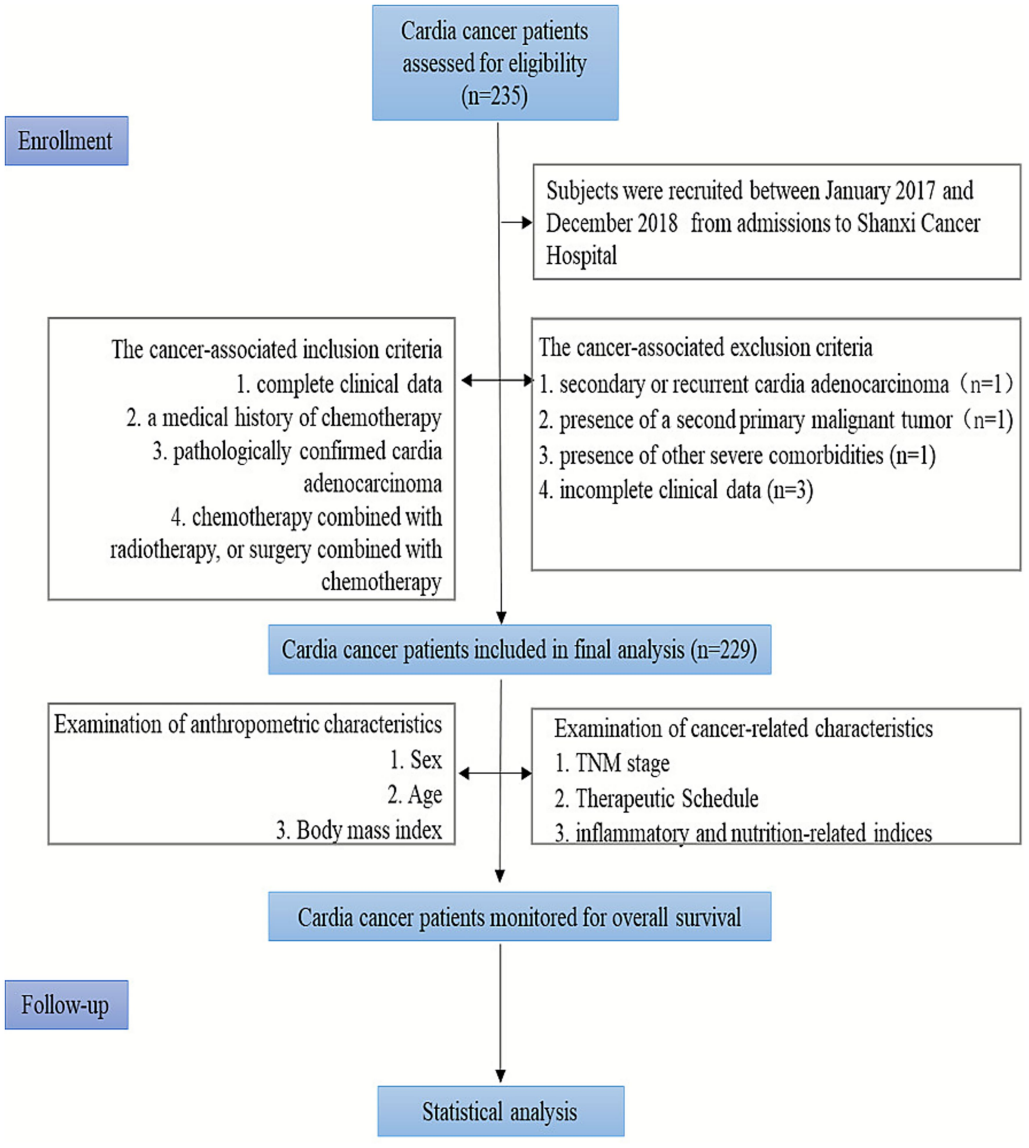
The flowchart of patient selection.

Data were collected from admissions to Shanxi Cancer Hospital between January 2017 and December 2018, comprising pre-treatment clinically relevant information for 229 patients with cardia cancer. Inclusion criteria: complete clinical data; pathologically confirmed cardia adenocarcinoma; a medical history of chemotherapy, chemotherapy combined with radiotherapy, or surgery combined with chemotherapy. Exclusion criteria: secondary or recurrent cardia adenocarcinoma; presence of a second primary malignant tumor; presence of other severe comorbidities; incomplete clinical data (Figure 1). This study was approved by the Ethics Committee of Shanxi Cancer Hospital (KY2023148).

### 2.2 Data collection

Data on patients’ sex, age, treatment regimen, TNM stage, BMI, and laboratory findings, including neutrophil count, lymphocyte count, platelet count, albumin levels, and erythrocyte distribution width, were collected. TNM staging was determined based on the American Joint Committee on Cancer 8th edition. The formulas for the indices are as follows: BMI = weight (kg) / height (m)^2^; PLR = platelet count (×10^9^/L) / lymphocyte count (×10^9^/L): HRR = hemoglobin (g/L) / width of erythrocyte distribution (fl); PNI = serum albumin (g/L) + 5 × lymphocyte count (×10^9^/L); HALP = hemoglobin (g/L) × serum albumin (g /L) × lymphocyte count (×10^9^/L) / platelet count (×10^9^/L). Treatment modalities in this study included chemotherapy, chemotherapy combined with radiotherapy, and surgery combined with chemotherapy. Among the chemotherapy regimens, SOX consisted of oxaliplatin 130 mg/m^2^ (static, day 1) and Tegio 40 mg/m^2^ (oral, days 1–14); XELOX included oxaliplatin 130 mg/m^2^ (static, day 1) and capecitabine 1,000 mg/m^2^ (oral, days 1–14). Radiotherapy was applied to cardia lesions and metastatic lymph nodes using 6 MV-X-ray intensity-modulated radiotherapy, with a dose of 50.4–60.0 Gy (1.8–2.0 Gy per dose, once daily, five times per week). The surgical procedure involved radical cardia cancer surgery.

### 2.3 Follow-up

This was primarily conducted through hospitalization, outpatient review, and telephone callbacks to clarify patient survival status. Overall Survival (OS) was defined as the time from the diagnosis of cardia cancer to the patient’s death or the follow-up cutoff date. The follow-up cut-off date was December 31, 2023, and survival was measured in months.

### 2.4 Statistical analysis

SPSS 26.0 software was used for all statistical analyses. The chi-square (X^2^) test was applied to count data between groups. X-title 3.6.1 software was used to determine the optimal critical values of BMI, PLR, HRR, PNI, and HALP score. The Kaplan–Meier method was employed for survival analysis, with the Log-rank test used to assess variability in survival time between different groups. Univariate and multivariate Cox proportional hazards regression analyses were conducted to evaluate the prognostic value of each variable. The receiver operating characteristic (ROC) curve was plotted, and the area under the curve (AUC) was calculated to evaluate the predictive value of each index for the prognosis of cardia cancer patients. A scoring system was developed based on the hazard ratios (HR), categorizing patients into low-risk, intermediate-risk, high-risk, and very high-risk groups using quartiles. Survival analysis was conducted among the groups. A *p*-value of < 0.05 was considered statistically significant.

## 3 Results

### 3.1 Clinical characteristics

A total of 229 patients with cardia cancer were included in this study, comprising 196 males and 33 females. The patients’ ages ranged from 43 to 82 years, with a median age of 64 years. Among them, 72 patients were aged < 60 years, and 157 patients were aged ≥ 60 years. The distribution of TNM stages was as follows: six patients in Stage I, 22 patients in Stage II, 94 patients in Stage III, and 107 patients in Stage IV. Treatment modalities included 165 patients receiving chemotherapy, nine patients receiving radiotherapy and chemotherapy, and 55 patients undergoing surgery combined with chemotherapy (Table 1).

**Table 1:** Baseline patient clinicopathological characteristics.

Clinical characteristics	*N* (%)
Sex	
Male	196 (85.59)
Female	33 (14.41)
Age (years)	
<60	72 (31.44)
≥60	157 (68.56)
TNM stage	
I + II	28 (12.23)
III + IV	201 (87.77)
Therapeutic schedule	
Chemotherapy	165 (72.05)
Chemotherapy + radiotherapy	9 (3.93)
Surgery + chemotherapy	55 (24.02)
BMI (kg/m^2^)	
<20.43	96 (41.92)
≥20.43	133 (58.08)
PLR	
<140.59	98 (42.79)
≥140.59	131 (57.21)
HRR	
<2.85	96 (41.92)
≥2.85	133 (58.08)
PNI	
<49.98	125 (54.59)
≥49.98	104 (45.41)
HALP score	
<28.23	82 (35.81)
≥28.23	147 (64.19)

### 3.2 Optimal cut-off values for BMI, PLR, HRR, PNI, and HALP score

In the 229 patients, the optimal cut-off values for BMI, PLR, HRR, PNI, and HALP score were 20.43 kg/m^2^, 140.59, 2.85, 49.98, and 28.23, respectively. According to the optimal cut-off values, the distribution of inflammation and nutrition-related indexes was characterized by BMI < 20.43 kg/m^2^ in 96 cases and BMI ≥ 20.43 kg/m^2^ in 133 cases; PLR < 140.59 in 98 cases and PLR ≥ 140.59 in 131 cases; HRR < 2.85 for 96 cases and HRR ≥ 2.85 for 133 cases; PNI < 49.98 for 125 cases and PNI ≥ 49.98 for 104 cases; HALP score < 28.23 for 82 cases and HALP score ≥ 28.23 for 147 cases (Table 1).

### 3.3 Prognosis of patients based on sex, age, stage, treatment regimen, and pretreatment inflammatory and nutrition-related markers

**Figure 2:**
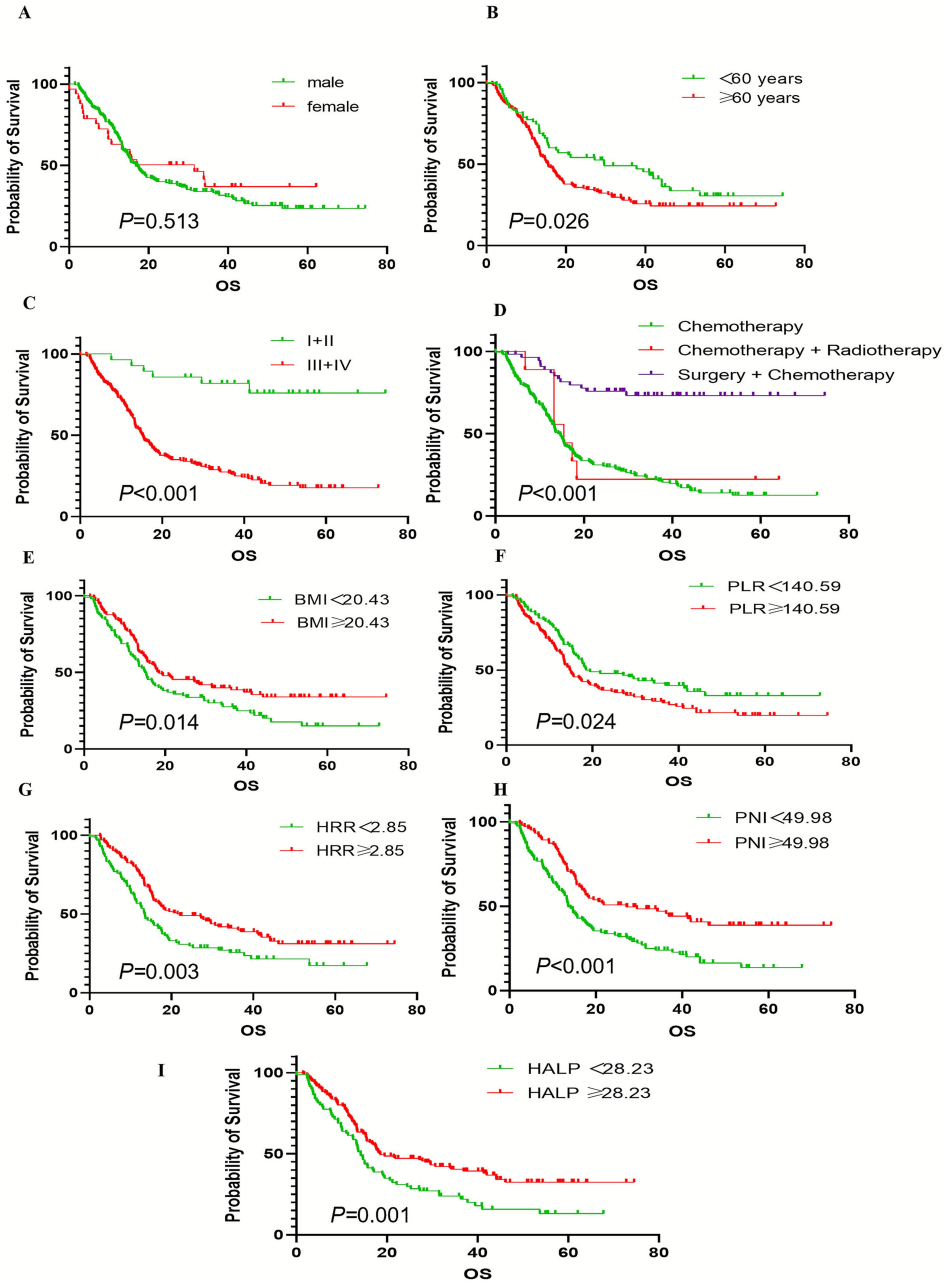
Kaplan–Meier estimates of the OS of cardia cancer according to the clinical characteristics. **(A)** The survival curves of OS between different sex groupings. **(B)** The survival curves of OS between different age groupings. **(C)** The survival curves of OS between different TNM staging groupings. **(D)** The survival curves of OS between different treatment regimen groupings. **(E)** The survival curves of OS between different BMI groupings. **(F)** The survival curves of OS between different PLR groupings. **(G)** The survival curves of OS between different HRR groupings. **(H)** The survival curves of OS between different PNI groupings. **(I)** The survival curves of OS between different HALP score groupings.

Kaplan–Meier survival analysis showed that age < 60 years, stage I/II, comprehensive treatment with surgical intervention, BMI ≥ 20.43 kg/m^2^, PLR < 140.59, HRR ≥ 2.85, PNI ≥ 49.98, and HALP score ≥ 28.23 were significantly correlated with longer overall survival (*p* < 0.05). However, there was no significant correlation between sex and OS (*p* > 0.05) (Figure 2).

### 3.4 Univariate and multivariate cox regression analysis

Cox univariate analysis showed that age < 60 years, stage I/II, comprehensive treatment with surgical involvement, BMI ≥ 20.43 kg/m^2^, PLR < 140.59, HRR ≥ 2.85, PNI ≥ 49.98 and HALP score ≥ 28.23 were associated with the prognosis of patients with cardia cancer (*p* < 0.05) (Table 2). The results of multivariate Cox analysis revealed that age, TNM staging, treatment regimen, HRR, and PNI were independent prognostic factors affecting the prognosis of patients with cardia cancer. Specifically, patients aged < 60 years, those receiving comprehensive treatment with surgical involvement, those in stage I/II, and those with high HRR and high PNI were associated with longer overall survival (Table 3).

**Table 2:** Univariate Cox regression analysis for overall survival in patients with cardia cancer.

Clinicopathological	*N*	Single factor analysis	Statistical value	*p* value
		HR (95%CI)		
Sex				
Male	33	1		
Female	196	1.17 (0.73–1.90)	0.43	0.513
Age (years)				
<60	72	1		
≥60	157	1.49 (1.05–2.13)	7.6	0.006
TNM stage				
I + II	28	1		
III + IV	201	9.05 (3.35–24.49)	78.82	<0.001
Therapeutic schedule				
Surgery + chemotherapy	55	1		
Chemotherapy	165	5.15 (2.96–8.94)	41.73	<0.001
Chemotherapy + radiotherapy	9	4.09 (1.65–10.16)	10.51	0.002
BMI (kg/m^2^)				
<20.43	96	1		
≥20.43	133	0.68 (0.49–0.93)	6.04	0.014
PLR				
<140.59	98	1		
≥140.59	131	1.45 (1.05–2.01)	5.11	0.024
HRR				
<2.85	96	1		
≥2.85	133	0.62 (0.45–0.85)	9.03	0.003
PNI				
<49.98	125	1		
≥49.98	104	0.50 (0.36–0.70)	17.11	<0.001
HALP rating				
<28.23	82	1		
≥28.23	147	0.59 (0.43–0.82)	10.41	0.001

**Table 3:** Cox regression analysis for overall survival in patients with cardia cancer.

Clinicopathological	*N*	Multivariate analysis	Statistical value	*P* value
		HR (95%CI)		
Age (years)				
<60	72	1		
≥60	157	1.48 (1.03–2.12)	4.40	0.036
TNM stage				
I + II	28	1		
III + IV	201	3.04 (1.23–7.52)	5.78	0.016
Therapeutic schedule				
Surgery + chemotherapy	55	1		
Chemotherapy	165	3.23 (1.76–5.95)	14.17	<0.001
Chemotherapy + radiotherapy	9	2.83 (1.09–7.32)	4.58	0.032
HRR				
≥2.85	133	1		
<2.85	96	1.62 (1.16–2.28)	7.83	0.005
PNI				
≥49.98	104	1		
<49.98	125	1.62 (1.15–2.30)	7.40	0.007

### 3.5 Prognostic risk stratification analysis

**Figure 3:**
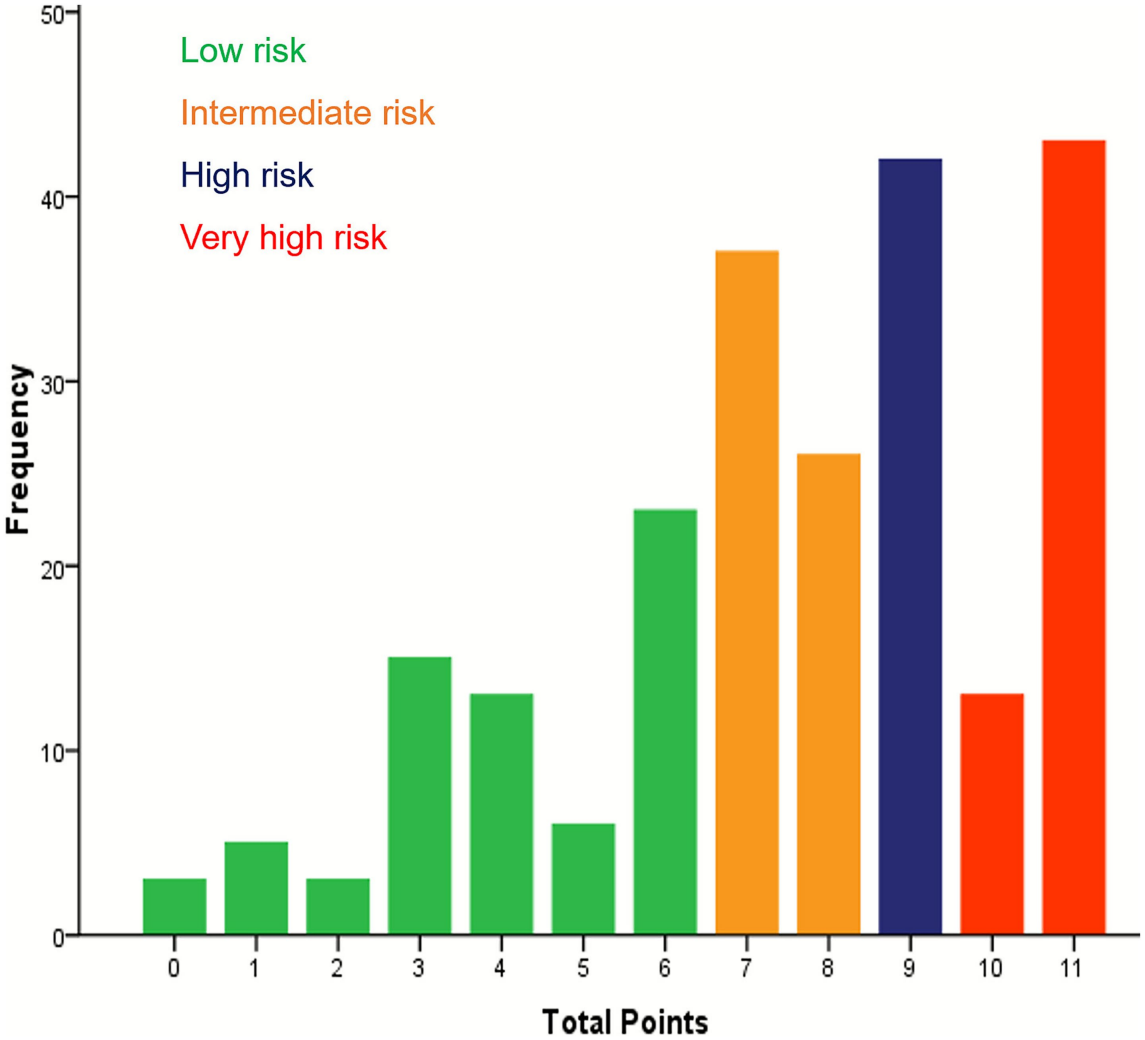
Showing the distribution of patients across the total sum of points and four risk groups in cardia cancer patients.

**Figure 4:**
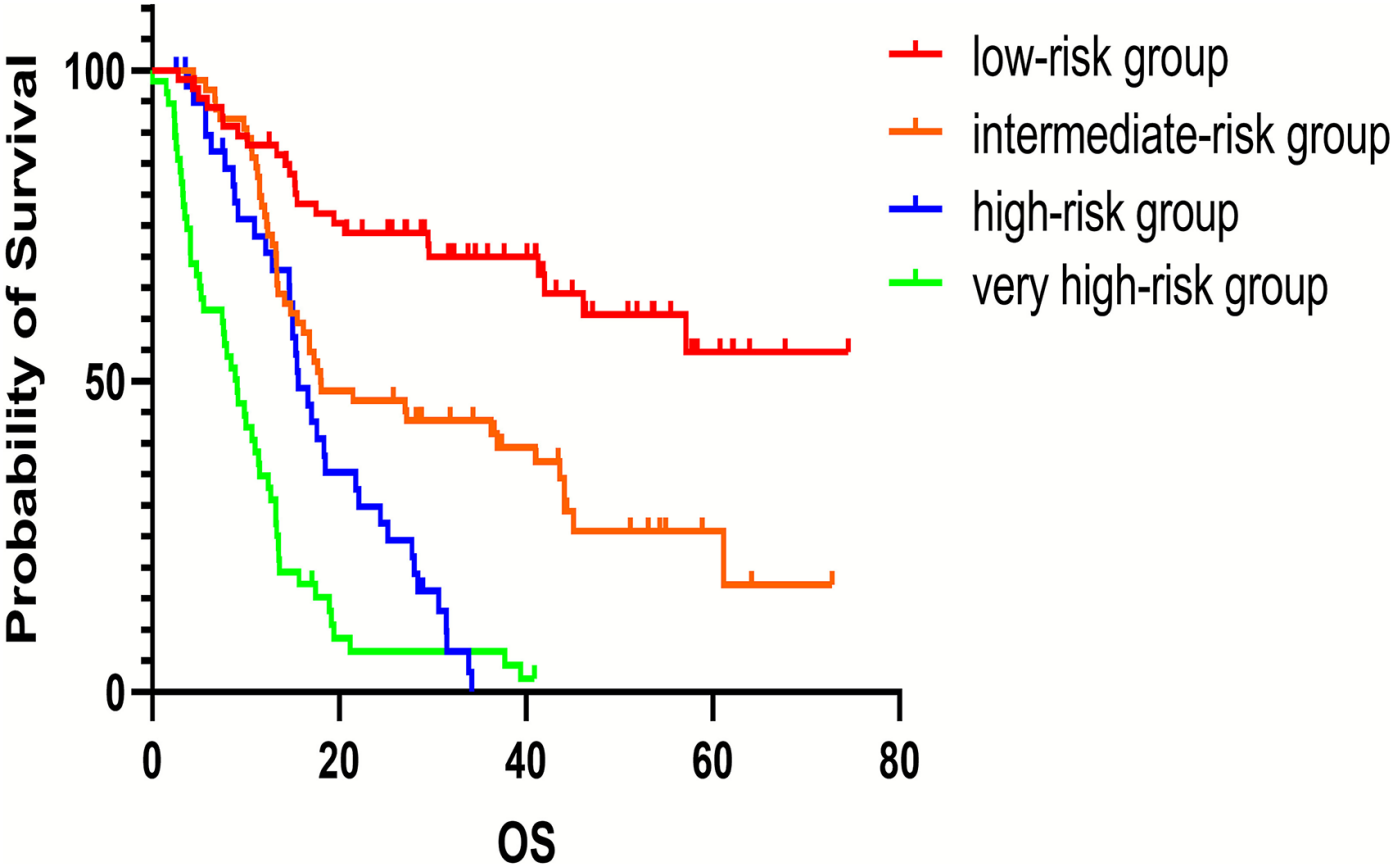
The survival curves for cardia cancer patients by risk group. The low-risk group had the most favorable prognosis, whereas the very high-risk group had the worst prognosis. The differences among the four groups were statistically significant (*p* < 0.05).

According to the multifactorial Cox regression analysis, the five variables that significantly predicted the prognosis of patients with cardia cancer were age over 60 years, III/IV (TNM stage), absence of surgical intervention in the treatment regimen, HRR below 2.85, and PNI below 49.98. These five variables were assigned points based on their rounded HRs. Age ≥ 60 years was assigned one points, TNM staging, chemotherapy and chemotherapy combined with radiotherapy were each assigned three points, and HRR < 2.85 and PNI < 49.98 were assigned two points (Table 4). An increase in the total score was associated with decreased OS among patients with cardia cancer. Based on quartiles of the score distribution, patients were classified into four groups: low-risk (0–6 points), intermediate-risk (7–8 points), high-risk (9 points), and very high-risk (10–11points) (Figure 3). The median survival durations for patients in the low-risk, intermediate-risk, high-risk, and very high-risk groups were undefined, 18.05 months, 15.63 months, and 9.10 months, respectively, with statistically significant differences observed among the groups (*p* < 0.05) (Figure 4).

**Table 4:** The variables were assigned points based on HRs.

Variable	HR	95%CI	Weight
Age ≥ 60 (years)	1.48	1.03–2.12	1
Staging (III/IV)	3.04	1.23–7.52	3
Chemotherapy	3.23	1.76–5.95	3
Chemotherapy + radiotherapy	2.83	1.09–7.32	3
HRR < 2.85	1.62	1.16–2.28	2
PNI < 49.98	1.62	1.15–2.30	2

### 3.6 Predictive value of prognostic risk stratification with cardia cancer patients

ROC curve analysis revealed that prognostic risk stratification had a superior predictive value compared with age, TNM stage, treatment regimen, HRR, and PNI (Figure 4).

**Figure 5:**
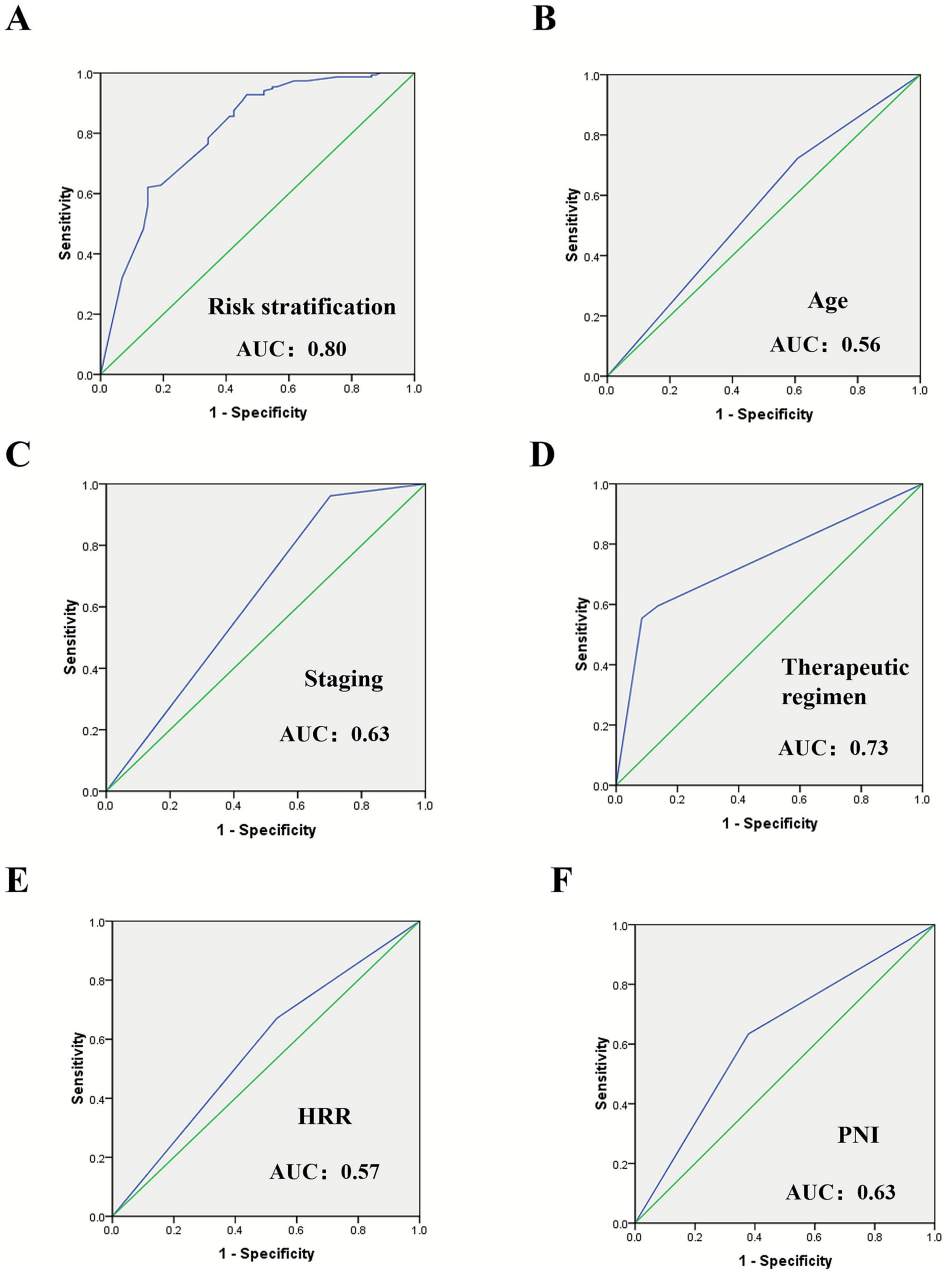
The ROC curves for predicting OS in cardia cancer patients based on prognostic risk stratification, age, TNM stage, treatment regimen, HRR and PNI. The prognostic risk stratification demonstrated the best predictive performance. **(A)** The ROC curves for predicting OS in cardia cancer patients based on risk stratification, **(B)** The ROC curves for predicting OS in cardia cancer patients based on age, **(C)** The ROC curves for predicting OS in cardia cancer patients based on staging, **(D)** The ROC curves for predicting OS in cardia cancer patients based on therapeutic regimen, **(E)** The ROC curves for predicting OS in cardia cancer patients based on HRR, **(F)** The ROC curves for predicting OS in cardia cancer patients based on PNI.

The AUC were 0.80 (95% CI: 0.74 ~ 0.87), 0.56 (95% CI: 0.48 ~ 0.64), 0.63 (95% CI: 0.55 ~ 0.71), 0.73 (95% CI: 0.66 ~ 0.81), 0.57 (95%CI: 0.49 ~ 0.65), and 0.63 (95%CI: 0.55 ~ 0.71), respectively (Figure 5).

## 4 Discussion

Gastric cancer is categorized into cardia and non-cardia cancer. Cardia cancer refers to mucosal tumors located within 3 cm below and 2 cm above the gastroesophageal junction line (1). Due to its unique anatomical location and nonspecific clinical symptoms, cardia cancer is often diagnosed at advanced stages, leading to a shortened survival period. Consequently, identifying effective predictive indicators is of particular importance. Nutritional status, which is closely associated with the prognosis of cancer patients, has garnered increasing attention from oncologists (6–8). Building on inflammatory indexes, the integration of nutrition-related indexes to predict the prognosis of solid tumors, such as gastric cancer, has shown promising potential (9–11). Nonetheless, studies exploring the correlation between the combination of these tests and the prognosis of cardia cancer remain limited.

Inflammation-related biomarkers such as C-reactive protein (CRP), neutrophil-to-lymphocyte ratio (NLR), and PLR demonstrate significant associations with malignant tumors (12–14). However, CRP and NLR are inherently non-specific, which often limits their ability to accurately reflect tumor-related inflammation status (15). In contrast, PLR exhibits relative stability to short-term inflammatory fluctuations, potentially offering superior prognostic value for cancer patients (15). The study innovatively integrated multiple pre-treatment parameters, including PLR, HRR, PNI, and HALP score, with clinical characteristics of cardia cancer patients to overcome the limitations of a single indicator and enhance the accuracy of predicting prognosis in cardia cancer patients.

Univariate analysis revealed that patients younger than 60 years, those with stage I/II disease, those receiving comprehensive treatment including surgery, and those with high BMI, low PLR, high HRR, high PNI, and high HALP score had better prognoses. Multivariate Cox analysis further identified age, TNM stage, treatment regimen, HRR, and PNI as independent factors influencing the prognosis of patients with cardia cancer.

Relevant studies have highlighted the significant role of age in predicting survival outcomes in patients with tumors, including cardia cancer (16, 17). The results of the present study are consistent with them.

TNM staging is a well-established indicator for assessing the prognosis of patients with cardia cancer. However, increasing evidence suggests that patients with the same TNM stage can have markedly different prognoses (18). This discrepancy may stem from the fact that TNM staging, which is primarily based on imaging findings, does not account for the intrinsic biological differences among patients. Combining TNM staging with biologically relevant indexes, such as inflammation and nutrition-related indicators, may provide a more comprehensive reflection of these differences. The prediction model developed in this study highlights the potential of integrating TNM staging with inflammatory and nutritional markers to enhance the accuracy of prognostic predictions for patients with cardia cancer.

The symptoms of cardia cancer are often subtle, resulting in a lower proportion of early-stage patients. The comprehensive treatment is the primary modality. The findings of the present study demonstrate that patients experienced longer survival periods with treatment regimens involving surgery. Surgery continues to be the cornerstone of curative treatment for gastric cancer (19). It also reflects the importance of local treatment modalities for cardia cancer patients. Chemotherapy combined with radiotherapy did not show statistical significance compared to chemotherapy alone, which may be attributed to the small sample size.

Patients with low hemoglobin levels could indicate that tumor cell clones may possess greater biological invasiveness and that the patient may have poorer nutritional status (20, 21). Of note, Kunishige et al. (22) previously analyzed clinical data from 801 patients with gastric cancer and found that preoperative anemia was significantly associated with poorer prognoses. Red cell distribution width (RDW), a marker reflecting tumor-related inflammatory responses, has also been linked to poor outcomes in malignant tumors (23, 24). Studies by Petrella et al. (25) and Shota et al. (26) confirmed that preoperative RDW is significantly associated with the prognosis of gastric cancer patients. HRR, defined as the ratio of Hb to RDW, is a novel marker (27) that provides an integrated assessment of the inflammatory and nutritional status of tumor patients. The present study’s findings suggest that a high HRR, characterized by higher Hb and lower RDW levels, is associated with better prognosis in patients with cardia cancer.

PNI, calculated from serum albumin levels and lymphocyte counts, is a well-established indicator for assessing nutritional and immune status and predicting risk in malignant tumors, which has been extensively used to evaluate various solid tumors’ treatment outcomes (28–30). In gastric cancer, PNI is widely applied in assessing surgical risk and the efficacy of neoadjuvant therapy (31). Moreover, it serves as an independent predictor of postoperative complications and survival outcomes (32). Herein, our results indicate that higher PNI values are associated with longer survival in patients with cardia cancer.

The prognostic risk stratification model for cardiac cancer, incorporating age, TNM stage, treatment regimen, HRR, and PNI, effectively categorized patients into low-, intermediate-, high-, and very high-risk groups, with statistically significant differences in survival among these groups. The integrated predictive performance of the model (AUC = 0.80) was significantly superior to that of any single indicator, demonstrating that the combination of multiple parameters provides a more accurate prognostic assessment. In clinical practice, the publicity and education on early diagnosis of cardiac cancer should be strengthened, and strive for surgical opportunities through neoadjuvant. Additionally, timely and continuous monitoring of biomarkers such as HRR and PNI should be emphasized. Although this study did not incorporate post-treatment data, the multivariate analysis confirmed the stability and predictive power of the baseline indicators, thereby offering valuable insights for clinical risk stratification and the formulation of targeted intervention strategies.

Nonetheless, given that this is a retrospective study, and due to differences in sample size, patient selection criteria, and laboratory testing methods, the optimal cut-off values of predictive indicators varied across the relevant studies, leading to potential bias. Therefore, future multi-center, large-sample clinical studies are needed to validate the combined value of conventional and emerging indicators, and explore the potential of pre-treatment nutritional interventions and other therapies to modulate these indicators.

## Data Availability

The original contributions presented in the study are included in the article/supplementary material, further inquiries can be directed to the corresponding authors.
